# Dimensions of childhood adversity differentially affect biological aging in major depression

**DOI:** 10.1038/s41398-022-02198-0

**Published:** 2022-10-04

**Authors:** Ryan Rampersaud, Ekaterina Protsenko, Ruoting Yang, Victor Reus, Rasha Hammamieh, Gwyneth W. Y. Wu, Elissa Epel, Marti Jett, Aarti Gautam, Synthia H. Mellon, Owen M. Wolkowitz

**Affiliations:** 1 Weill Institute for Neurosciences and Department of Psychiatry and Behavioral Sciences, University of California San Francisco (UCSF) School of Medicine, San Francisco, CA USA; 2 University of California San Francisco (UCSF) School of Medicine, San Francisco, CA USA; 3 Medical Readiness Systems Biology, Center for Military Psychiatry and Neuroscience, Walter Reed Army Institute of Research, Silver Spring, MD USA; 4 Headquarters, Walter Reed Army Institute of Research, Silver Spring, MD USA; 5 Department of OB-GYN and Reproductive Sciences, UCSF School of Medicine, San Francisco, CA USA

**Keywords:** Epigenetics in the nervous system, Depression

## Abstract

Adverse childhood experiences have been consistently linked with physical and mental health disorders in adulthood that may be mediated, in part, via the effects of such exposures on biological aging. Using recently developed “epigenetic clocks”, which provide an estimate of biological age, several studies have demonstrated a link between the cumulative exposure to childhood adversities and accelerated epigenetic aging. However, not all childhood adversities are equivalent and less is known about how distinct dimensions of childhood adversity relate to epigenetic aging metrics. Using two measures of childhood adversity exposure, we assess how the dimensions of *Maltreatment* and *Household Dysfunction* relate to epigenetic aging using two “second-generation” clocks, GrimAge and PhenoAge, in a cohort of unmedicated somatically healthy adults with moderate to severe major depression (*n* = 82). Our results demonstrate that the dimension of *Maltreatment* is associated with epigenetic age acceleration (EAA) using the PhenoAge but not the GrimAge clock. This association was observed using both the Childhood Trauma questionnaire (CTQ; β = 0.272, *p* = *0.013*) and the Adverse Childhood Experiences (ACEs) questionnaire (β = 0.307, *p* = *0.005*) and remained significant when adjusting for exposure to the dimension of *Household Dysfunction* (β = 0.322, *p* = *0.009*). In contrast, the dimension of *Household Dysfunction* is associated with epigenetic age deceleration (β = *−0.194, p* = *0.083*) which achieved significance after adjusting for exposure to the dimension of *Maltreatment* (β = *−0.304, p* = *0.022*). This study is the first to investigate these effects among individuals with Major Depressive Disorder and suggests that these dimensions of adversity may be associated with disease via distinct biological mechanisms.

## Introduction

A wealth of evidence suggests that exposure to childhood adversity is associated with increased risk of chronic somatic disease [1–3], premature mortality [4], and psychopathology in adulthood [5, 6], including Major Depressive Disorder (MDD). While the biological mechanisms linking childhood adversity to MDD, morbidity [7, 8] and mortality have not been fully elucidated several studies have suggested that modulation of biological aging (as assessed by telomere length [9–12], mitochondrial function [13], and pubertal timing [14]), is one mechanism by which childhood adversity is embedded into the individual and contributes to both somatic and mental health consequences in adulthood.

More recently, studies utilizing “epigenetic clocks” [15–17] have demonstrated the link between exposure to childhood adversity and biological aging. To date, several DNA methylation-based epigenetic clocks have been developed using a variety of methods, with the “first-generation” clocks (Horvath [18] and Hannum [19]) trained to predict chronological age and the “second generation” clocks (PhenoAge [20] and GrimAge [21]) trained to predict health outcomes and time to death. The deviation between an individuals’ chronological age and epigenetic age can be used to calculate relative epigenetic age acceleration or epigenetic age deceleration and has been widely studied in a variety of somatic [22–26] and psychiatric conditions [11, 27–29] including studying the effects of childhood adversity [16, 30, 31].

Using the “cumulative risk” model [32, 33], which simply tallies the number of adversities experienced to create a cumulative risk score, several studies have demonstrated a link between the number of adversities experienced and epigenetic age acceleration. A more recent model, the “dimensional model” of adversity and psychopathology [32, 34–37] hypothesizes that individual adverse experiences may be condensed into dimensions of experience which share common pathophysiological mechanisms.

Historically, exposures assessed using the Adverse Childhood Experiences (ACEs) questionnaire (as we do in this study) have been grouped into two categories: *Maltreatment* and *Household Dysfunction* [38, 39] and later work confirmed these dimensions as explanatory factors [40]. These dimensions differentiate between exposures that are directed towards the child (*Maltreatment;* which includes all forms of abuse and neglect) versus those that affect the child “indirectly” via their environment (*Household Dysfunction;* including exposures such as living in a household with substance use, mental illness, etc.*)*. While no studies to date have examined the relationship between these dimensions and biological aging metrics, some studies have suggested they may have unique patterns of association with psychiatric symptoms [41].

Several dimensions of adversity may exist and a model from McLaughlin and colleagues [32] hypothesized that adversity could be categorized into the dimensions of *Threat* (which includes exposure to abuse and reflects potential harm to the individual) and *Deprivation* (which includes experiences of physical/emotional neglect as well as food insecurity and cognitive deprivation early in life). Prior work by Sumner et al. [42] in a cohort of children/adolescents demonstrated that exposure to threat-related adversity was associated with accelerated epigenetic aging (using the Horvath clock) as well as pubertal stage [42]. In contrast, exposure to deprivation-related adversity had the opposite effect and was associated with decelerated biological aging (as measured by pubertal stage but not epigenetic aging).

**Fig. 1:**
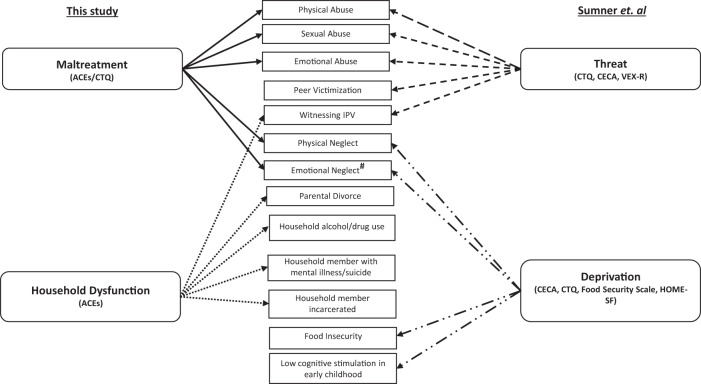
Comparison of dimensions of childhood adversity. Here we show dimensions of adversity assessed in this study (*Maltreatment/Household Dysfunction*) compared to the dimensions of *Threat/Deprivation* as assessed by Sumner et al. In parentheses, we show the scales utilized to create these composite scores. Individual items incorporated into each of these domains are shown in the center with arrows indicating to which dimensions they belong. *Threat* and *Deprivation* have been assessed using a multi-modal approach, while our study assessed exposures with the commonly utilized Childhood Trauma Questionnaire (CTQ) and Adverse Childhood Experiences (ACEs) questionnaire. We observed that *Threat* and *Maltreatment* are highly overlapping with the major differences being inclusion of neglect items and exclusion of witnessing IPV item into the dimension of *Maltreatment*. Peer victimization (PV) was not assessed in this study as we did not utilize the Violence Exposure Scale-Revised (VEX-R) which provides a measure of different types of peer victimizations experiences. *Household Dysfunction* and *Deprivation* were assessed utilizing different scales. ^#^Emotional neglect was assessed in distinct ways between these studies. Specifically, the ACEs/CTQ assesses subjective appraisals of emotional neglect. In prior studies which utilized the *Threat/Deprivation* framework, emotional neglect was specifically assessed using the Childhood Experiences of Care and Abuse (CECA) which assesses neglectful behaviors (rather than an appraisal of the distant experience of neglect). Cognitive stimulation was assessed using Home Observation Measurement of the Environment (HOME-SF).

Recent work has demonstrated that exposure to childhood adversity is associated with greater epigenetic aging in adults with MDD compared to those without a history of such exposures [43]. However, there have been no studies to date which have specifically investigated how distinct dimensions of childhood adversity are associated with epigenetic aging in adults with MDD. While there are some overlapping features between the dimensions of *Threat/Deprivation* with *Maltreatment/Household Dysfunction* they are not identical (Fig. 1). We assess the dimensions of *Maltreatment/Household Dysfunction* in this study given the use of the CTQ and ACEs questionnaire from which we cannot re-create the dimensions of *Threat/Deprivation*. Here, we test the hypothesis that amongst adults with MDD, the dimensions of *Maltreatment* and *Household Dysfunction* differentially affect biological aging as measured by two “second-generation” epigenetic clocks, PhenoAge and GrimAge, which are capable of capturing variations in the risk of disease and death.

## Methods

### Ethics statement

This research was approved by the Institutional Review Board of the University of California, San Francisco.

### Recruitment procedures and study participants

One hundred subjects with MDD were recruited by flyers, bulletin board notices, Craigslist postings, newspaper ads, and clinical referrals and informed consent was obtained from all subjects. All subjects were diagnosed with MDD without psychotic symptoms according to the Structured Clinical Interview for DSM-IV-TR Axis I Disorders (SCID), which was the version in use at the beginning of this study, and diagnosis was verified by clinical interview with a board-certified psychiatrist. Depressive symptomatology was evaluated with the 17-item Hamilton Score for Depression Rating Scale (HDRS), with a current score of ≥17 being an inclusion criterion. Depressed subjects were excluded for presence of the following: bipolar disorders, psychotic symptoms, history of psychosis outside of a mood episode, any eating disorder or post-traumatic stress disorder (PTSD) within one month of entering the study, and substance abuse or dependence (including alcohol) within six months of entering the study. The study participants had no acute illnesses/infections, chronic inflammatory disorders, neurological disorders, or other major medical condition. All subjects were free of psychotropic medications, including antidepressants, and other potentially interfering medications and had not had any vaccinations for at least 6 weeks prior to enrollment in the study, and none was taking vitamin supplements above the US recommended daily allowances. Short-acting sedative-hypnotics were allowed as needed for sleep up to a maximum of 3 times per week, but none within 1 week prior to blood draws. Prior to each study visit, all subjects had to pass a urine toxicology screen for drugs of abuse and a urine test for pregnancy for women of child-bearing potential.

### DNA preparation and analysis of methylation

Blood samples were drawn in the morning following an overnight fast. Whole blood was collected in acid citrate dextrose tubes for preparation of DNA. Aliquoted samples were stored frozen at −80 °C until use. DNA was extracted from whole blood using QIAmp DNA purification kits (Qiagen, Redwood City, CA), followed by quality check using a Tapestation (Agilent). Identification and analysis of methylated CpGs used protocols used by our group previously [44]. Genomic DNA (500 ng) was treated with sodium bisulfite using the Zymo EZ96 DNA Methylation Kit (Zymo Research, Orange, CA, USA), and genome-wide DNA methylation patterns were profiled using the Infinium HumanMethylation450 BeadChip array (Illumina, Inc., San Diego, CA, USA). Noob background correction [45] was used to pre-process the data prior to submitting it to the DNAm Age website https://dnamage.genetics.ucla.edu for analysis [18].

### Childhood exposure to adversity

Early life adversity was measured using two scales, the Childhood Trauma Questionnaire (CTQ) [46] and the ACE-10 questionnaire [47]. The CTQ is a validated self-report questionnaire that assesses five types of maltreatment between birth and 18 years: sexual, physical, and emotional abuse as well as emotional and physical neglect [46]. Items are rated on a 5-point scale from “Never True” to “Very Often True”. In order to assess the effects of types of maltreatment we created a composite *Abusive Maltreatment* score (sum of scores on sexual, physical, and emotional abuse subscales) and a composite *Neglectful Maltreatment* score (sum of scores on physical and emotional neglect subscales). A total *All Maltreatment* (total CTQ) score was created by summing the *Abusive Maltreatment* score and the *Neglectful Maltreatment* score [48]. The ACE scale was also used to assess early life adversity, where items are rated based on their absence (score = 0) or presence (score = 1). This scale consists of 10 items: five items for subtypes of maltreatment (physical, sexual, or emotional abuse, physical or emotional neglect which is similar to the total CTQ score) and five items measuring a distinct domain of experience, not assessed by the CTQ, referred to as household dysfunction (parental separation or divorce, witnessing domestic violence, and incarceration, substance abuse, or mental illness of a household member) occurring between birth and 18 years. We create two composite scores based on prior studies [40, 49] which found a two-factor solution for the ACEs survey: Maltreatment, and Household Dysfunction. A total *Maltreatment* score (ACEs) was created by summing the first five items (physical, sexual, and emotional abuse as well as emotional and physical neglect) which is similar to the *All Maltreatment* score (Total CTQ score). A total *Household Dysfunction* score was created by summing the last five items (parental separation, incarceration, mental illness, witnessing IPV, and substance use) which is not assessed by the CTQ. A *Total* ACE score was created by summing the *Maltreatment* score and *Household Dysfunction* score (sum of all 10 items on the ACEs questionnaire). We utilized the CTQ as well as the ACE in our analyses for several reasons. First, the CTQ has been previously used in other studies to assess the effect of *Maltreatment* exposure [50] as well as abuse versus neglect [43, 48] (both forms of maltreatment) and we aimed to determine whether we could reproduce in MDD participants the results previously identified in non-depressed participants. Additionally, we include the ACEs questionnaire which not only assesses the domain of *Maltreatment* (identical to the exposures assessed in the CTQ), but also assesses the unique dimension of *Household Dysfunction*. Prior studies have suggested that these distinct domains can lead to specific kinds of psychopathology [51, 52], which may be mediated in part via distinct pathophysiological mechanisms. Creation of other composite scores is detailed in the Supplementary Material.

### Statistical analysis

Of the original 100 subjects with MDD recruited, four were excluded due to missing data for CTQ, ten were excluded due to lack of data on epigenetic aging measures (not assayed), two were excluded as they had initiated antidepressant treatment prior to their blood draw, one was excluded due to lack of data on household income (used as a covariate), and one was excluded due to missing data on smoking habits (used as a covariate; *n* = 82). Additionally, one subject was excluded in our analysis using the ACEs survey due to missing data on ACEs exposures (*n* = 81). PhenoAge and GrimAge acceleration—defined as the residuals resulting from regressing PhenoAge and GrimAge on chronological age (*AgeAccelPheno and AgeAccelGrim*, respectively)—were calculated for all participants. DNA methylation assays (described above) were carried out in two batches and epigenetic age acceleration variables were calculated within each batch. Linear regression models were used to determine the associations between the epigenetic clocks and *All Maltreatment* score as well as *Abusive Maltreatment* and *Neglectful Maltreatment* score. Separate linear regression models were used to identify associations between the epigenetic clocks and total ACE score, as well as *Maltreatment* and *Household Dysfunction* scores. Several a priori covariates were included in sequentially adjusted linear regression models including gender, race/ethnicity (White, Black, Latino, Other), BMI, smoking status (never, former, or current), household income (≤$49 000, $50 000-$99 999, $100 000-$200 000, or ≥$200 000) [53] and DNAm-based estimates of blood cell composition. Specifically, the DNAm-based estimates of blood cell composition included [54, 55] naïve CD8 T cells, CD8pCD28nCD45Ran “exhausted T cells”, B cells, CD4 T cells, NK cells, Monocytes, and Granulocytes (as calculated by the DNAm Age website [18]). In addition, to assess the unique associations between the domains of adversity and biological aging, we controlled for the other domains of adversity for each exposure. Specifically, for our analysis examining the relationship between *Abusive Maltreatment* and epigenetic aging we include *Neglectful Maltreatment* in the model and vice versa. In our analysis of the relationship between *Maltreatment* score (ACEs) and epigenetic aging, we include *Household Dysfunction* in the model and vice-versa. All data were assessed for normality prior to analyses and Blom transformation [56] was applied to *AgeAccelGrim* to achieve normality. Inspection of the data revealed that one value for *AgeAccelPheno* was approximately three standard deviations away from the mean *AgeAccelPheno*. To reduce disproportionate influence on the analysis of this point, the dataset was winsorized (using R package DescTools) and winsorized models are presented in our sensitivity analysis. Results did not differ appreciably from those of nonwinsorized data. We first carried out analyses with CTQ given that prior studies have examined associations between this measure of adversity and the “second-generation” clocks [48, 50] and subsequently assessed associations with the ACEs questionnaire.

## Results

### Participant characteristics

Our final sample included 82 individuals with MDD who had data on early life adversity (as measured by the CTQ) and both epigenetic age clocks and demographic variables used as covariates. One of these subjects was missing ACE data, leaving 81 subjects for those analyses; The mean age of the sample was 36.9 years and was 53% female. Additional participant characteristics are noted in Table 1.

**Table 1:** Participant characteristics.

**Sociodemographic characteristics**	
Age	37.1 (13.1) [20–68]
Female sex [n(%)]	44 (53.7)
*Race/Ethnicity*	
White	42 (51.2)
Black	8 (9.8)
Latino	8 (9.8)
Other	24 (29.3)
*Income [n(%)]*	
≤49,000	54 (65.9)
50,000–99,999	15 (18.3)
100,000–199,999	11 (13.4)
≥200,000	2 (0.024)
**Lifestyle characteristics**	
Body Mass Index	25.5 (4.2) [18.8–36.3]
*Smoking History [n(%)]*	
Never	44 (53.7)
Former	22 (26.8)
Current	16 (19.5)
**Characterization of Major Depressive Disorder**	
*D*epressive Symptoms Score (IDS)	25.1 (5.31) [12–37]
Hamilton Depression Rating Sclae (HDRS)	19.8 (3.28) [13–34]
Duration of Current Depressive Episode (days)	2258.3 (3663.8) [24–18536]
Chronicity of Lifetime Depression (months)	154.7 (145.8) [3.41–609]
Number of Depressive Episodes	3.59 (2.68) [1–12]
Lifetime Days of Depression	4707.8 (4438.5) [104–18536.1]
**Characterization of Adversity Exposure**	
*Childhood Trauma Questionnaire*	
* All Maltreatment Score* (Total CTQ)	42.5 (12.4) [25–82]
Physical Abuse	6.9 (2.3) [5–15]
Emotional Abuse	10.4 (4.3) [5–25]
Sexual Abuse	6.3 (3.5) [5–23]
Physical Neglect	6.9 (2.6) [5–15]
Emotional Neglect	12.1 (4.9) [5–23]
*Adverse Childhood Experiences Survey*	
Total ACE	2.8 (2.2) [0–10]
* Maltreatment Score*	1.4 (1.5) [0–5]
Yes	53 (65.4)
No	28 (34.6)
* Household Dysfunction Score*	1.4 (1.3) [0–5]
Yes	57 (70.3)
No	24 (29.7)

Mean (sd) [range] presented unless otherwise indicated.

### Abusive maltreatment but not neglectful maltreatment is associated with accelerated epigenetic aging

Using the CTQ, we examined the relationship between the *All Maltreatment* score (total CTQ score) and accelerated epigenetic aging, as has been previously done [42, 43, 48, 57]. We observed that this *All Maltreatment* score (which reflects exposure to all forms of abuse and neglect) was positively associated with *AgeAccelPheno* (*β* = *0.272 p* = *0.013*) but not *AgeAccelGrim* (*β* = *0.007 p* = *0.949*) (Table 2). This *All Maltreatment* score (total CTQ score) was not associated with depression severity (*β* = *−0.053 p* = *0.638*). Based on significant results between the *All Maltreatment* score and PhenoAge but not GrimAge, we examined *AgeAccelPheno* as our primary outcome but present the regression results for *AgeAccelGrim* for completeness. We next assessed whether *Abusive Maltreatment* and *Neglectful Maltreatment* were differentially associated with epigenetic aging. Notably, we observed that *Abusive Maltreatment* was positively associated with *AgeAccelPheno* (*β* = *0.288 p* = *0.009*) (Table 2) which survived adjustment for several covariates including blood cell composition. In contrast, *Neglectful Maltreatment* was not associated with accelerated epigenetic aging as measured by either clock (*β* = *0.187 p* = *0.093)*. (Table 2; Scatterplots are shown in Supplementary Fig. 1), even after adjustment for several covariates. We observed that *Abusive Maltreatment* and *Neglectful Maltreatment* scores were significantly correlated with each other (*r* = 0.570, *p* < 0.001), consistent with co-occurrence of adversity types. Given this co-occurrence, we evaluated the specific effects of these composite scores by including both forms of adversity in the model. The effect size for the *Abusive Maltreatment* score was somewhat reduced when controlling for co-occurring *Neglectful Maltreatment* score *(β* = *0.265 p* = *0.068)*, while the effect size for the *Neglectful Maltreatment* score was greatly reduced when controlling for the *Abusive Maltreatment* score (*β* = *0.050 p* = *0.709)*, suggesting that abuse was the main contributor to the association between the *All Maltreatment* score and accelerated epigenetic aging.

**Table 2:** Associations of *Maltreatment* (CTQ) with biological aging.

	*AgeAccelPheno*			*AgeAccelGrim*		
	*b* (95% CI)	β	*p*-value	*b* (95% CI)	β	*p*-value
*All Maltreatment*						
Model 1	0.12 [0.026, 0.214]	0.272	**0.013**	0.0006 [−0.017, 0.018]	0.007	0.949
Model 2	0.122 [0.017, 0.226]	0.277	**0.023**	0.0008 [−0.016, 0.017]	0.011	0.92
*Abusive Maltreatment*						
Model 1	0.208 [0.054, 0.362]	0.288	**0.009**	0.008 [−0.021, 0.037]	0.061	0.586
Model 2	0.212 [0.038, 0.386]	0.293	**0.018**	0.003 [−0.024, 0.031]	0.025	0.816
Model 3^a^	0.192 [−0.014, 0.398]	0.265	*0.068*	0.005 [−0.028, 0.038]	0.0398	0.752
*Neglectful Maltreatment*						
Model 1	0.157 [−0.027, 0.341]	0.187	*0.093*	−0.009 [−0.042, 0.025]	−0.057	0.61
Model 2	0.153 [−0.042, 0.349]	0.182	0.122	−0.001 [−0.031, 0.029]	−0.007	0.943
Model 3^b^	0.042 [−0.184, 0.269]	0.05	0.709	−0.004 [−0.040, 0.032]	−0.027	0.821

Model 1. Unadjusted model.

Model 2. Adjusted for gender, BMI, smoking status, race, household income and DNAm-based markers of immune cell composition.

^a^Model 3. Model 2 further adjusted for *Neglectful Maltreatment* score.

^b^Model 3. Model 2 further adjusted for *Abusive Maltreatment* score.

### Household dysfunction is associated with decelerated epigenetic aging

Next, we utilized the ACEs questionnaire to assess the effects *Maltreatment* (identical to the total CTQ score) and *Household Dysfunction* on epigenetic aging [58]. First, we assessed the relationship between the *Total ACE* score (which combines *Maltreatment* and *Household Dysfunction* scores) and epigenetic aging (Table 3). *Total ACE* score was not significantly related to either *AgeAccelPheno* (*β* = *0.091 p* = *0.421)* or *AgeAccelGrim* (*β* = *0.032 p* = *0.778)*.

**Table 3:** Associations of *ACE* scores *(Total, Maltreatment, Household Dysfunction)* with Biological Aging.

	*AgeAccelPheno*			*AgeAccelGrim*		
	*b* (95% CI)	β	*p*-value	*b* (95% CI)	β	*p*-value
*Total ACE score*						
Model 1	0.230 [−0.336, 0.797]	0.091	0.421	0.015 [−0.088, 0.117]	0.032	0.778
Model 2	0.165 [−0.520, 0.850]	0.065	0.632	−0.020 [−0.122, 0.082]	−0.044	0.697
*Maltreatment*						
Model 1	1.16 [0.353, 1.96]	0.307	**0.005**	.0005 [−0.147, 0.157]	0.008	0.944
Model 2	1.05 [0.123, 1.97]	0.277	**0.027**	−0.04 [−0.185, 0.099]	−0.063	0.552
Model 3^a^	1.21 [0.309, 2.12]	0.322	**0.009**	−0.044 [−0.190, 0.101]	−0.065	0.544
*Household Dysfunction*						
Model 1	−0.820 [−1.75, 0.111]	−0.194	*0.083*	0.034 [−0.137, 0.204]	0.044	0.697
Model 2	−1.06 [−2.18, 0.071]	−0.249	*0.066*	0.005 [−0.167, 0.178]	0.007	0.95
Model 3^b^	−1.29 [−2.38, −0.194]	−0.304	**0.022**	0.014 [−0.161, 0.189]	0.018	0.875

Note the opposite direction of association between epigenetic age acceleration and maltreatment or household dysfunction score.

Model 1. Unadjusted model.

Model 2. Adjusted for gender, BMI, smoking status, race, household income and DNAm-based markers of immune cell composition.

^a^Model 3. Model 2 further adjusted for *Household Dysfunction* score.

^b^Model 3. Model 2 further adjusted for *Maltreatment* score.

Most individuals (*n* = 37) endorsed both *Maltreatment* and *Household Dysfunction* experiences, while a smaller number endorsed only *Maltreatment* experiences (*n* = 16) or only *Household Dysfunction* experiences (*n* = 20) and a minority reporting no exposures at all (n = 8). Similar to the *All Maltreatment* score (total CTQ score), the ACEs *Maltreatment* score was positively associated with *AgeAccelPheno* (*β* = *0.307 p* = *0.005)* but not *AgeAccelGrim* (*β* = *0.008 p* = *0.944)* (Table 3; Scatterplots are shown in Supplementary Fig. 2). Maltreatment measures (CTQ and ACEs) were highly correlated (*r* = *0.701 p* < *0.0001*). Given the co-occurrence of *Maltreatment* and *Household Dysfunction (r* = *0.230, p* = *0.039)*, we controlled for the dimension of *Household Dysfunction* and observed that the relationship between the ACEs *Maltreatment score* and *AgeAccelPheno* remained significant. Further analyses revealed that depressed individuals with an ACEs *Maltreatment* score of one or more (“*Any*”) showed greater epigenetic age acceleration as measured by *AgeAccelPheno*, than individuals who had a score of zero (“*None*”; *p* = 0.014*, Cohen’s d* = 0.6*;* Supplementary Table 1). This remained significant after controlling for the co-occurring *Household Dysfunction* score.

In contrast, the *Household Dysfunction* score was negatively associated with *AgeAccelPheno* after controlling for the effects of co-occurring *Maltreatment* (*β* = *−0.304 p* = *0.022)*, suggesting that *Household Dysfunction* is associated with decelerated epigenetic aging and may have negated the effect of *Maltreatment* when examining the *Total ACE* score (Table 3). Further analyses revealed that individuals with a *Household Dysfunction* score of one or greater (“*Any”*) showed reduced epigenetic age acceleration compared to individuals with a score of zero (“*None”*; *p* = 0.008*, Cohen’s d* = 0.6; Supplementary Table 2). These group differences persisted after controlling for co-occurring *Maltreatment* score. No significant group differences were observed with *AgeAccelGrim* (data not shown).

While we utilized the validated two-factor solution of *Maltreatment* and *Household Dysfunction* to identify differential associations with epigenetic aging, some studies have suggested that witnessing interpersonal violence (IPV) loads on to the dimension of *Maltreatment* rather than *Household Dysfunction* [49, 59]. We created a modified *Maltreatment* score which included IPV and removed it from the *Household Dysfunction* score (Supplementary Methods). The modified *Maltreatment* score was positively associated with *AgeAccelPheno* after controlling for the modified *Household Dysfunction score* (*β* = *0.317 p* = *0.013*). Additionally, the modified *Household Dysfunction* score was negatively associated with *AgeAccelPheno* after controlling for the modified *Maltreatment score* (*β* = *−0.327 p* = *0.012;* Supplementary Table 3). The effect sizes were larger using the modified *Household Dysfunction* score, compared to the initial model [45, 56].

### Associations between childhood adversity and first-generation epigenetic clocks

Given that exposure to childhood adversity has been associated with premature morbidity/mortality [60], our primary analyses examined the association between these exposures and the “second generation” clocks. However, we also explored associations between childhood adversity and two “first-generation” epigenetic clocks (Horvath and Hannum). We found no significant associations between *Maltreatment* scores (CTQ and ACE) or *Household Dysfunction* scores and epigenetic age acceleration with either clock (Supplementary Table 4 and Supplementary Table 5).

### Sensitivity analysis

To ensure that our results were not due to the undue influence of potential outliers, winsorized models are also presented. We continue to observe that the *Abusive Maltreatment* score was associated with accelerated epigenetic aging as measured by *AgeAccelPheno* while the *Neglectful Maltreatment* score showed no association (Supplementary Table 6, Supplementary Table 7, Supplementary Fig. 3). Additionally, we continue to see that the *Household Dysfunction* score was associated with decelerated epigenetic aging as measured by *AgeAccelPheno* (Supplementary Table 8, Supplementary Table 9, Supplementary Fig. 4). Group differences between depressed individuals exposed to *Maltreatment* or *Household Dysfunction* compared to non-exposed depressed individuals persisted in the winsorized models (Supplementary Table 10 and Supplementary Table 11).

## Discussion

Exposure to childhood adversity has been associated with the development of physical [61] and mental health issues in adulthood [6, 62, 63] and may be mediated, in part, via effects on biological aging. Here, we assess how different dimensions of childhood adversity are associated with epigenetic aging amongst unmedicated somatically healthy individuals with moderate to severe MDD using two recently developed metrics of epigenetic aging-PhenoAge and GrimAge. We demonstrate that greater experiences of maltreatment (driven by the abusive, but not neglectful, forms of maltreatment) are associated with accelerated epigenetic aging (as measured by PhenoAge but not GrimAge) in adulthood. In contrast, the experience of household dysfunction is associated with decelerated epigenetic aging, as measured by the PhenoAge clock. These results provide support for the idea that childhood adversity encompasses distinct dimensions of experience that may be associated with vulnerability to disease via unique biological mechanisms.

To date, a single study has examined the association of childhood adversity exposure with epigenetic aging in adults with Major Depressive Disorder. The work by Han et al. [43] utilized an epigenetic clock developed to gauge chronological age to demonstrate that amongst adults with MDD, exposure to childhood trauma was associated with greater epigenetic aging. Our results build on these findings to demonstrate that in addition to “dose,” the “type” of adversity is an important factor which has distinct associations with epigenetic aging. Furthermore, we demonstrate these associations using a “second-generation” clock which stands out for its ability to capture biological disturbances associated with aging. Although we focus here on these “second-generation” clocks, we did explore associations between childhood adversity and “first-generation” clocks (Horvath and Hannum clocks) and found no significant associations between adversity measures and epigenetic aging. Possible reasons for the lack of association are discussed further below.

Our work adds to a body of literature studying the effects of childhood adversity on epigenetic aging in adults without psychiatric diagnoses. Our results provide further support for a dimensional approach in studying the effects of childhood adversity on outcomes later in life. Recently, McCrory et al. [64] demonstrated that childhood adversity (specifically poverty) resulted in approximately 1.2 to 2 years accelerated epigenetic aging as measured by the GrimAge and Pace of Aging clock, although it is unclear how their assessment of childhood poverty relates to our measure of *Household Dysfunction*. Additionally, Hamlat et al. [48] demonstrated that childhood adversity (measured by the CTQ) was associated with accelerated epigenetic aging using the GrimAge clock (but not with the PhenoAge, Horvath, or Hannum clocks). Similar to our own results (although with GrimAge and not PhenoAge), they demonstrated that this association was driven by the experience of abuse rather than neglect. Reasons for the differential associations with epigenetic clocks are discussed below.

The 10 ACEs assessed in our study have typically been conceptualized into the two constructs of *Maltreatment* and *Household Dysfunction* [38, 39] and this factor structure has been confirmed in large scale studies [40]. While these types of experiences differ on the basis of whether they are “directly” experienced (*Maltreatment*) or “indirectly” experienced (*Household Dysfunction*), how this might lead to opposite patterns of association with epigenetic aging is unclear. We can speculate that directly experienced adversities represent exposures which are more endangering to the individual while those which are indirectly experienced (i.e., household member incarceration, drug use, mental illness, etc.) may reflect limited access to resources. These findings can be understood in the context of life history theory [65, 66] which suggests that the effects of these exposures on aging are evolutionary adaptations. Those which directly affect and thus endanger the individual (*Maltreatment*) results in accelerated aging as a way of prioritizing reproduction. In contrast, those that are indirectly experienced in the environment of the individual and are associated with limited access to resources and care (*Household Dysfunction*) results in decelerated aging in order to delay reproduction until favorable conditions arise.

While the biological underpinnings of this differential association are not clear, studies looking at outcomes have shown that these exposures may have differential associations with psychiatric symptoms [41, 52]. These findings suggest that these exposures could alter unique biological pathways. Notably, the work of Ridout et al. [67] has attempted to understand how exposure to resource poor conditions and disruption of the maternal-infant bond (which we hypothesize resembles the dimension of *Household Dysfunction*) might contribute to decelerated aging. Using a non-human primate model, they demonstrate that exposure to such conditions in childhood resulted in longer telomeres in adulthood, which may be connected via increased production of Glucagon-Like Peptide-1 (GLP-1). Additional studies are needed to better understand the biological basis for the differential effects of these exposures.

Given that we cannot create true *Threat/Deprivation* scores given the use of the CTQ/ACEs questionnaire, our results are not directly comparable to the findings of Sumner et al. [42]. However, the pattern of associations observed with *Maltreatment* and *Household Dysfunction* mirrors the findings observed with the dimensions of *Threat* and *Deprivation* [32, 34–36, 42, 68]. Given this, our results suggest that *Maltreatment* reflects threat-related exposure and is associated with accelerated epigenetic aging. While the *Maltreatment* score incorporates some deprivation-related exposures (subjective recall of emotional and physical neglect), our results suggest that the associations between *Maltreatment* and accelerated epigenetic aging is driven by *Abusive Maltreatment* rather than *Neglectful Maltreatment* and thus shows an association with aging consistent with a threat-related exposure. The lack of an expected association between *Neglectful Maltreatment* and epigenetic aging may be due to the possibility that these items (emotional/physical neglect) do not capture the full range of deprivation experiences. Prior studies examining the experience of deprivation included not only physical/emotional neglect but also food insecurity and low cognitive stimulation.

We hypothesize that the experiences captured by the *Household Dysfunction* score more fully captures deprivation-related experiences. This score assesses experiences such as living in a home with household members experiencing substance use, incarceration, divorce, etc. and may reflect early environments in which there is a lack of expected inputs, neglect, as well as limited cognitive stimulation early in life [36]. Notably, when we removed the IPV item from the *Household Dysfunction* score and incorporated this into the *Maltreatment* score, we observed larger effect sizes. Witnessing IPV has been conceptualized as a threatening experience and this finding further supports the idea that *Household Dysfunction* reflects deprivation based exposures. Unfortunately, we were unable to create *Threat* and *Deprivation* scores due to methodological differences in how adversity was measured [35, 42, 69, 70].

While a wealth of evidence has shown associations between childhood adversity and epigenetic aging amongst a variety of populations, no single epigenetic clock has shown a consistent association with childhood adversity exposure. We hypothesize that this is related to differences in the study populations assessed, assessments of childhood adversity used, as well as the clocks reported on in each study. We note that Han et. al. examined associations between trauma and epigenetic aging in a community-based sample (distinct from our depressed cohort) and utilized a clock of their own derivation (distinct from the method employed in this study). While our results show associations between the *All Maltreatment* (Total CTQ) score and PhenoAge, we note that Hamlat et al. observed similar associations with GrimAge. While speculative, this could be because their study specifically examined aging in a cohort of stressed female caregivers without MDD whose overall trauma exposure was lower than that of the depressed participants in this study. Additionally, their cohort of subjects was observed to be older than the average age of individuals in the current study and not necessarily medication free. Furthermore, our results contrast from those of the TILDA study which found associations between childhood poverty and the GrimAge clock. We note that this study assessed these markers in individuals aged 50–87 years old. While it is not clear how this would translate into associations with different clocks, we note that each clock is unique with regards to their derivation methods and it has been noted that there is little overlap in methylation sites contained within the epigenetic clocks [20, 71]. It is conceivable that the experience of adversity may differentially affect aging processes captured by these methylation sites in depressed versus non-depressed populations and with varying degrees of adversity. Additionally, it is possible that the associations observed with GrimAge in these studies is related to the advanced age and/or possible somatic dysfunction/medical co-morbidity of these subjects. Thus, changes in epigenetic aging may be better captured by the GrimAge clock in these cohorts given its superior ability to predict time to death and may be poorly suited to the younger, medically healthy cohort utilized in this study.

Strengths of our study include the use of rigorously diagnosed, moderate to severely depressed individuals. We also note that our sample is unique in that it specifically recruited individuals who were not treated with medications (including antidepressants), which may exert epigenetic effects and confound epigenetic age assessments [72]. Additionally, our study incorporated only somatically healthy individuals with MDD which limits the effect of medical co-morbidity on epigenetic age assessment.

Our study has several limitations including our modest sample size and predominantly Caucasian sample, which requires our results to be validated in a larger, more diverse cohorts. Additionally, given our retrospective assessment of childhood adversity, we cannot assess the timing or duration of the reported exposures. Our study was unable to account for childhood SES which may be an important covariate. While SES may be associated with increased risk for experiencing threat and deprivation, it may also be associated with other unknown toxic exposures that may also contribute to altered epigenetic aging. Furthermore, we note that these results are specific to individuals with MDD and studies in other populations will be needed. In addition, we do not assess any psychological, behavioral, or biological mediators which may mediate the effects of childhood adversity on epigenetic aging. Given methodological differences, we cannot assess the dimensions of *Threat* and *Deprivation* as prior studies have and future studies should utilize a multi-modal approach to measurement of childhood adversity to create multiple dimensions of adversity and understand the relationships between these dimensions.

Taken together, these findings demonstrate that amongst healthy, unmedicated individuals with Major Depressive Disorder, dimensions of adversity can become embedded into the host in distinct ways that predict biological aging. Specifically, experiences of maltreatment (driven by abuse) contribute to accelerated epigenetic aging while experiences of household dysfunction contribute to decelerated epigenetic aging. Future studies should further investigate how these distinct dimensions of adversity differentially affect epigenetic modifications to identify biological pathways that are affected. A greater appreciation of dimensions of adversity will provide greater understanding of the mechanisms by which childhood adversity is embedded into the individual and guide the development of novel interventions to limit the long-lasting consequences of childhood adversity.

## Supplementary information

Supplementary Methods

Supplementary Figure Lengeds

Supplementary Figure 1

Supplementary Figure 2

Supplementary Figure 3

Supplementary Figure 4

Supplemenary Table 1

Supplementary Table 2

Supplementary Table 3

Supplementary Table 4

Supplementary Table 5

Supplementary Table 6

Supplementary Table 7

Supplementary Table 8

Supplementary Table 9

Supplementary Table 10

Supplementary Table 11

## Data Availability

R code available by contacting the authors.
