# Supplementary material for: Dimensions of childhood adversity differentially affect biological aging in major depression
Source: Transl Psychiatry. 2022 Oct 4;12:431. doi: 10.1038/s41398-022-02198-0 (PMC9532396; doi:10.1038/s41398-022-02198-0)
Supplement: Supplementary file 1 — Supplementary Methods [file 41398_2022_2198_MOESM1_ESM.docx]

**Supplementary Methods**

***Creation of Maltreatment with IPV/Household Dysfunction without IPV scores***

While the two-factor solution utilized in this study incorporated the witnessing IPV item into *Household Dysfunction* score, this has also been conceptualized as a threatening experience and has been incorporated into the *Maltreatment* score. Thus, we created a modified *Maltreatment* score by summing the physical, emotional, and sexual abuse items as well as the witnessing IPV item. We also created a modified *Household Dysfunction* score by summing the same items contained in the original score but omitting the witnessing IPV item. These modified scores were used in sequentially adjusted linear regression models to assess relationships with *AgeAccelPheno*.

***Statistical Analysis of Group Differences in Mean AgeAccelPheno***

Group differences in mean *AgeAccelPheno* between individuals with no exposure to maltreatment (ACE *Maltreatment* score = 0; “None”) compared to those with any maltreatment exposures (ACE *Maltreatment* score ≥ 1; “Any”) were assessed using an independent samples t-test. Variances between groups were assessed via Levene’s Test and observed to be equal. We additionally covaried for the variables noted above in an ANCOVA analysis. Group differences in mean *AgeAccelPheno* between individuals with no exposure to household dysfunction (ACE *Household Dysfunction* score = 0; “None”) compared to those with any household dysfunction exposures (ACE *Household Dysfunction* score ≥ 1; “Any”) were also assessed using an independent samples t-test. Variances between groups were assessed via Levene’s Test and observed to be equal.
