# Supplementary material for: Dimensions of childhood adversity differentially affect biological aging in major depression
Source: Transl Psychiatry. 2022 Oct 4;12:431. doi: 10.1038/s41398-022-02198-0 (PMC9532396; doi:10.1038/s41398-022-02198-0)
Supplement: Supplementary file 2 — Supplementary Figure Lengeds [file 41398_2022_2198_MOESM2_ESM.docx]

**Supplementary Figure Legends**

**Supplementary Figure 1.** Associations between AgeAccelPheno and (A) *All Maltreatment Score* (Total CTQ score), (B) *Abusive Maltreatment Score* (CTQ Abuse subscale) (C) *Neglectful Maltreatment Score* (CTQ Neglect subscale). See Table 2 for significance.

**Supplementary Figure 2.** Associations between *AgeAccelPheno* and (A) Total ACE score, (B) *Maltreatment* score (sum of items 1 – 5 on ACEs questionnaire) and (C) *Household Dysfunction* score (sum of items 6 – 10 on ACEs questionnaire). See Table 3 for significance.

**Supplementary Figure 3.** Associations between AgeAccelPheno and (A) *All Maltreatment Score* (Total CTQ score), (B) *Abusive Maltreatment Score* (CTQ Abuse subscale) (C) *Neglectful Maltreatment Score* (CTQ Neglect subscale). See Supplementary Table 6 and 7 for significance. Winsorized model presented.

**Supplementary Figure 4.** Associations between *AgeAccelPheno* and **(A)** Total ACE score, **(B)** *Maltreatment* score (sum of items 1 – 5 on ACEs questionnaire) and **(C)** *Household Dysfunction* score (sum of items 6 – 10 on ACEs questionnaire). See Supplementary Table 8 and 9 for significance. Winsorized models presented.
