# Supplementary figures and images for: Dimensions of childhood adversity differentially affect biological aging in major depression
Source: Transl Psychiatry. 2022 Oct 4;12:431. doi: 10.1038/s41398-022-02198-0 (PMC9532396; doi:10.1038/s41398-022-02198-0)

**A**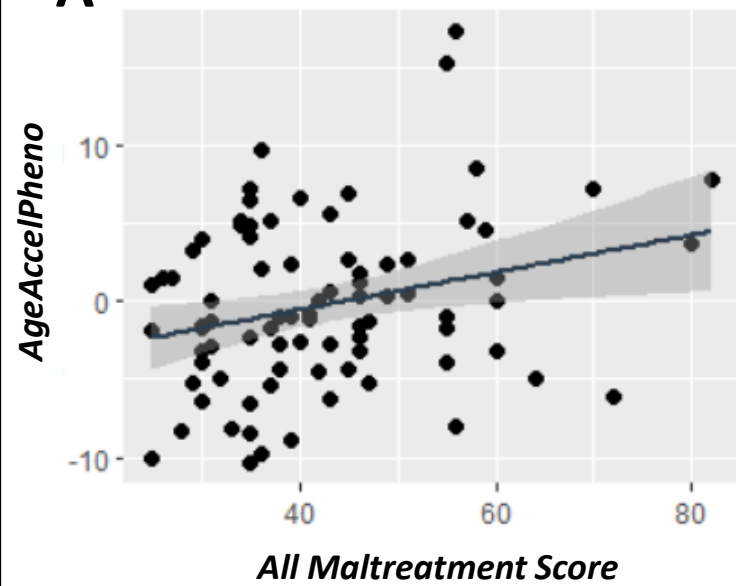**B**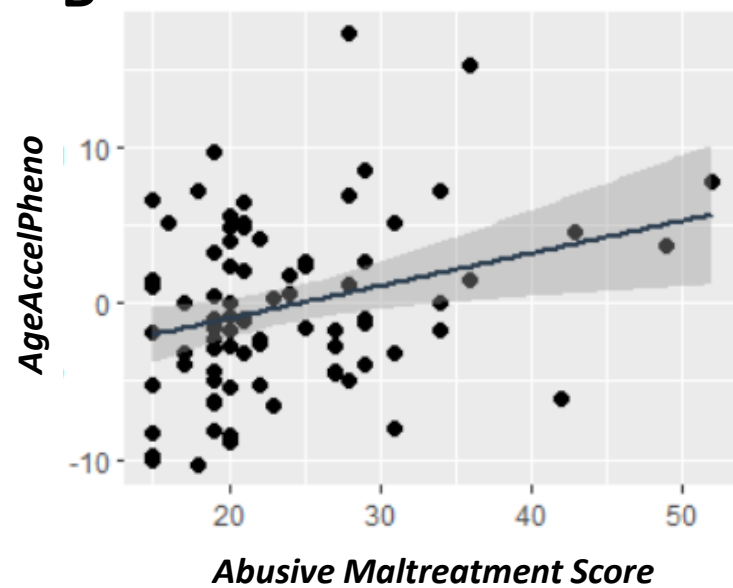**C**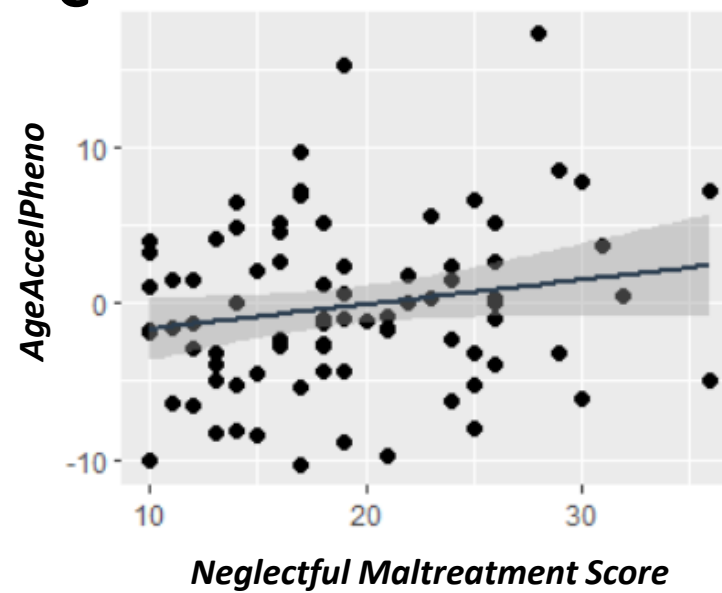

Supplement: Supplementary file 3 — Supplementary Figure 1 [file 41398_2022_2198_MOESM3_ESM.pdf]

**A**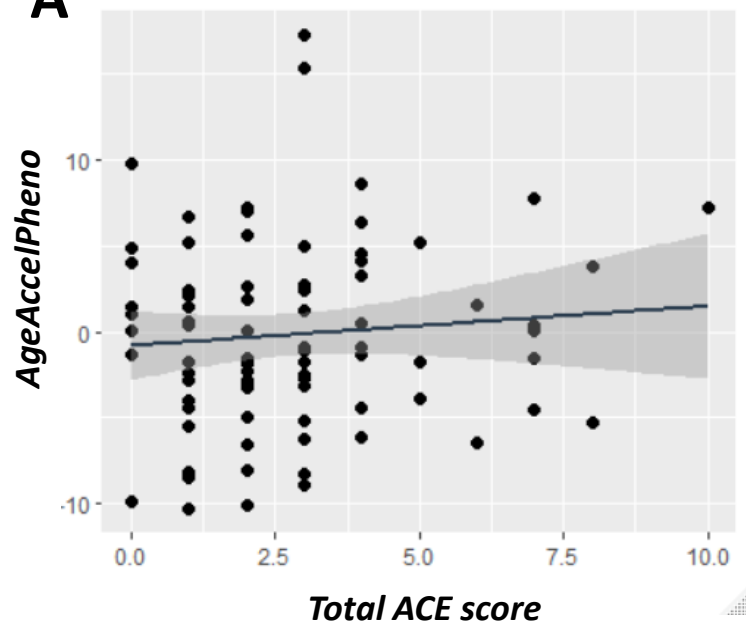**B**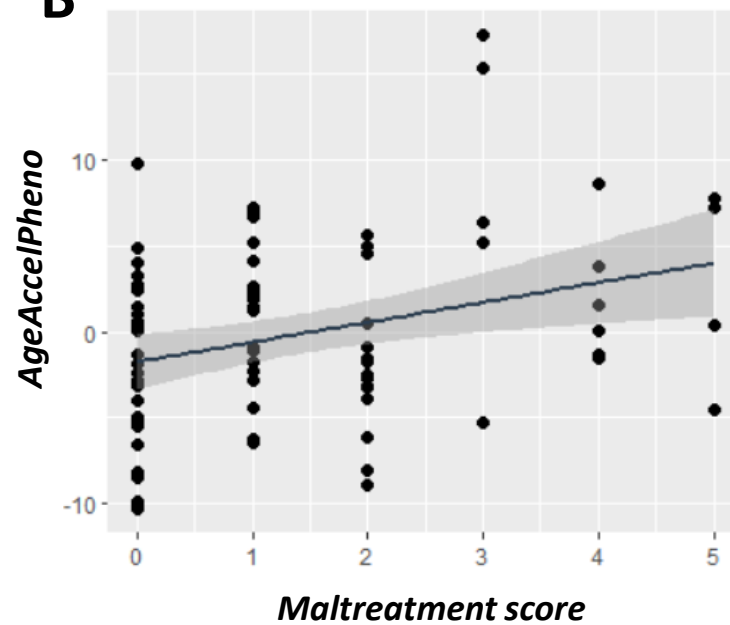**C**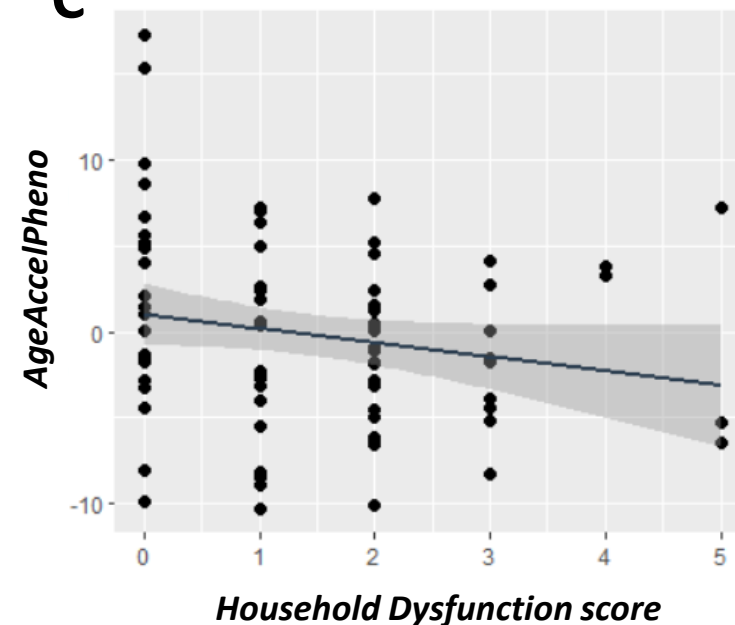

Supplement: Supplementary file 4 — Supplementary Figure 2 [file 41398_2022_2198_MOESM4_ESM.pdf]

**A**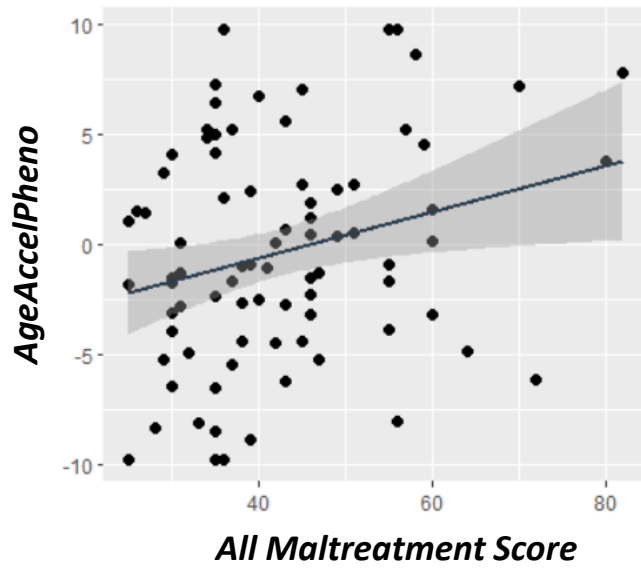**B**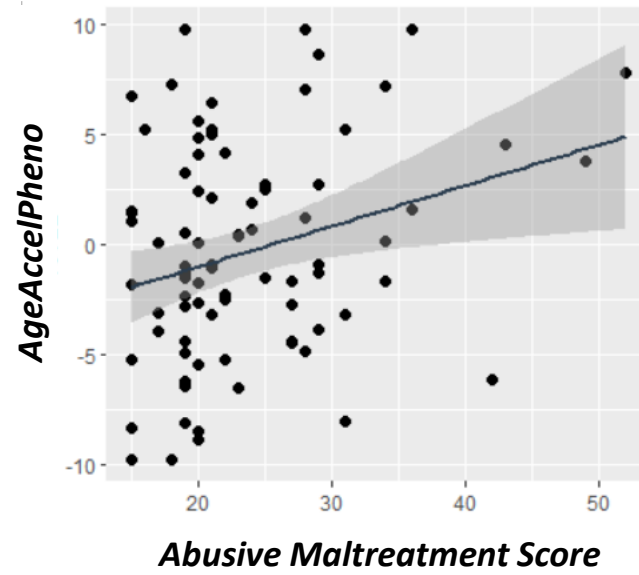**C**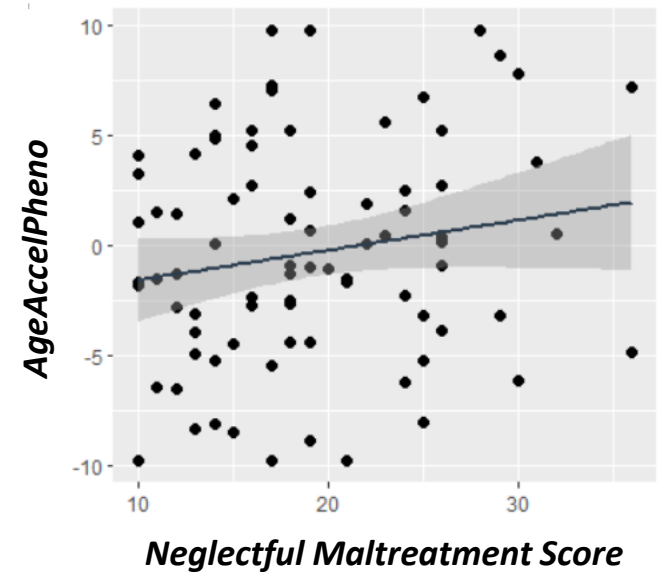

Supplement: Supplementary file 5 — Supplementary Figure 3 [file 41398_2022_2198_MOESM5_ESM.pdf]

**A**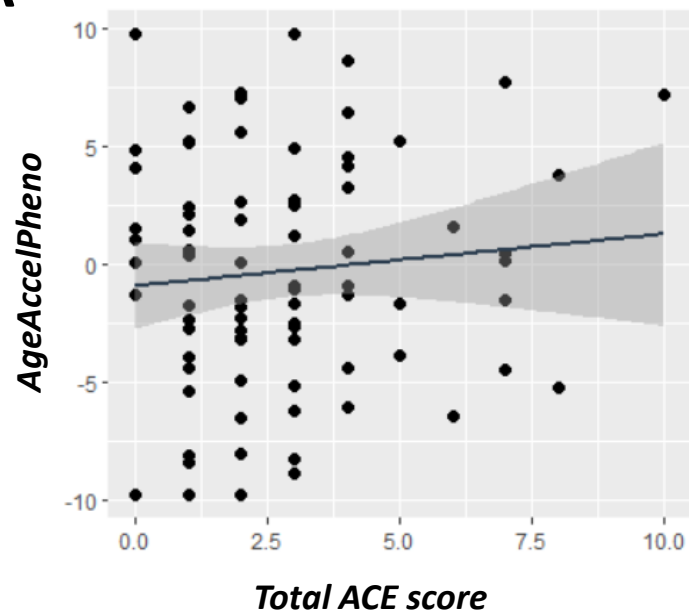**B**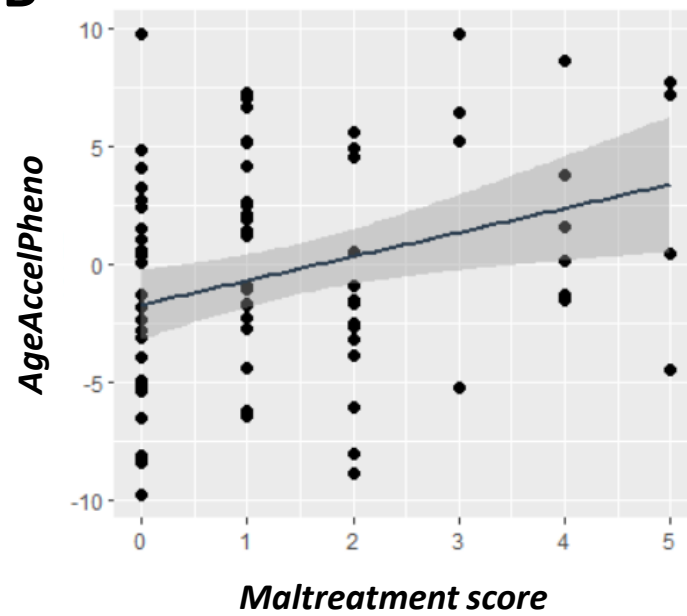**C**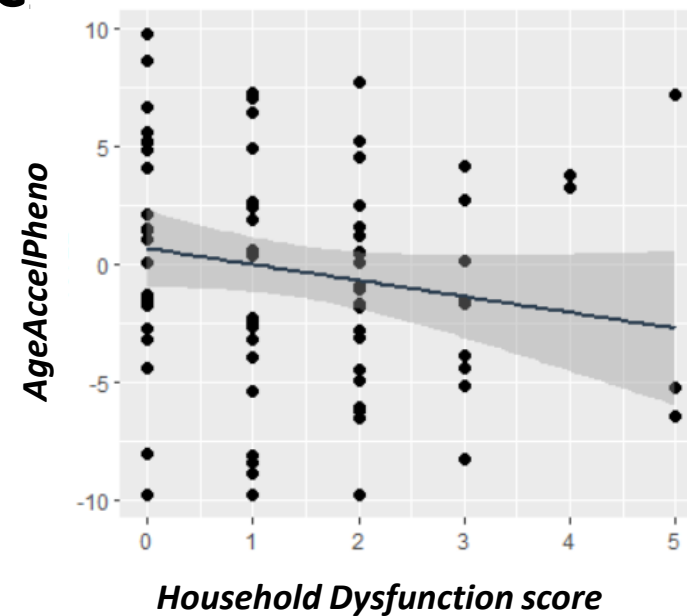

Supplement: Supplementary file 6 — Supplementary Figure 4 [file 41398_2022_2198_MOESM6_ESM.pdf]
